# Cost-effectiveness of Papillary Thyroid Microcarcinoma Treatment Pathways in Older Adults

**DOI:** 10.1093/geroni/igaf122.4091

**Published:** 2025-12-31

**Authors:** Rebecca Kowalski, Seth Rabinowitz, Julia Slejko, C Daniel Mullins, Yinin Hu

**Affiliations:** University of Maryland, Baltimore, Maryland, United States; University of Maryland, Baltimore, Maryland, United States; University of Maryland School of Pharmacy, Baltimore, Maryland, United States; University of Maryland School of Pharmacy, Baltimore, Maryland, United States; University of Maryland School of Medicine, Baltimore, Maryland, United States

## Abstract

Papillary thyroid microcarcinomas (PTMCs) are sub-centimeter cancers with an excellent prognosis. Cancer outcomes are similar across four common treatment pathways, making cost-effectiveness an important factor for treatment selection. In this decision analysis study, we hypothesized that compared to alternative strategies, active surveillance (AS) would be cost-effective for PTMC treatment among older adults. A Markov model was constructed to evaluate the incremental cost-effectiveness of three PTMC treatment pathways – radiofrequency ablation (RFA), partial thyroidectomy (PT), and total thyroidectomy (TT) – compared to AS. The base case was a 70-year-old patient with PTMC without high-risk features. Cost-effectiveness was estimated with a 10-year horizon and a willingness-to-pay threshold of $50,000/quality-adjusted life-year (QALY). Costs and outcome probabilities were derived from the literature. QALY weights for common PTMC-related health states were derived using time trade-off. Probabilistic sensitivity analysis (PSA) accounted for parameter uncertainty for QALY weights, AS & RFA disease progression probabilities, and treatment selection following disease progression. In this model, TT was strongly dominated by PT, and PT by RFA. RFA was cost-effective compared to AS, with an incremental cost-effectiveness ratio of $23,821/QALY. A 1-way sensitivity analysis demonstrated that RFA remained more cost-effective than AS for post-RFA disease progression rates up to 6.3 times that of AS. The PSA demonstrated RFA and AS were optimal in 58% and 36% of model iterations, respectively. In conclusion, for older adults with PTMC, RFA and AS are cost-effective relative to surgery; RFA is more cost-effective than AS, independent of its degree of disease progression risk reduction.

